# The importance of the concept and histological criteria of “intraepithelial squamous cell carcinoma” of the esophagus: in comparison between Western and Japanese criteria

**DOI:** 10.1007/s10388-017-0583-7

**Published:** 2017-06-14

**Authors:** Masayuki Itabashi, Anna Nasierowska-Guttmejer, Tadakazu Shimoda, Przemysław Majewski, Witold Rezner, Katarzyna Sikora, Ewa Śrutek, Katarzyna Stęplewska, Jarosław Swatek, Justyna Szumilo, Agnieszka Wierzchniewska-Ławska, Lech Wronecki, Ewa Zembala-Nożyńska, Tomio Arai, Masahiro Fujita, Hiroshi Kawachi, Masamitsu Unakami, Toshiro Kamoshida

**Affiliations:** 1 Pathology and Cytology Center, LSI Medience Corporation, 4-25-11 Azusawa, Itabashi-ku, Tokyo, 174-0051 Japan; 2 Department of Pathology, CSK MSW, Warsaw, Poland; 3 The National Cancer Research Center, Tokyo, Japan; 4 Department of Clinical Pathomorphology, Poznań University of Medical Sciences, Poznań, Poland; 5 Department of Pathology, Holycross Cancer Centre, Kielce, Poland; 6 Masovian Specialist Hospital in Radom, Radom, Poland; 7 Oncology Center in Bydgoszcz, Bydgoszcz, Poland; 8 Department of Pathomorphology, Medical University of Silesia, Zabrze, Poland; 9 Department of Clinical Pathomorphology, Medical University of Lublin, Lublin, Poland; 10 Department of Pathomorphology, Medical University of Łódź, Łódź, Poland; 11 Department of Tumor Pathology, M.Sklodowska-Curie Memorial Cancer Center and Institute of Oncology, Gliwice, Poland; 12 Department of Pathology, Tokyo Metropolitan Geriatric Hospital and Institute of Gerontology, Tokyo, Japan; 13 Department of Health Sciences, Nayoro City University, Hokkaido, Japan; 14 Department of Pathology, The Cancer Institute Hospital, Japanese Foundation for Cancer Research, Tokyo, Japan; 15 Pathology Division, Watari Hospital, Fukushima, Ibaraki-ken Japan; 16 Endoscopy Division and Department of Internal Medicine, Hitachi General Hospital, Hitachi, Ibaraki-ken Japan

**Keywords:** Noninvasive (intraepithelial) carcinoma, Esophageal squamous cell carcinoma, Western histological criteria, Japanese histological criteria, High/low-grade dysplasia

## Abstract

**Background:**

There are differences in the histological diagnostic criteria for early stage gastrointestinal carcinoma between Western and Japanese pathologists. Western histological criteria of carcinoma are “presence of stromal invasion of neoplastic cells”, while Japanese criteria are “the degree of cytological and structural abnormality of neoplastic cells, regardless of stromal invasion”. The aim of the present study is to clarify and review the present status of the Western and Japanese histological criteria of early stage esophageal squamous cell carcinoma (SCC) and also to clarify their significance and accuracy.

**Methods:**

Twenty-nine Polish, German, and Japanese pathologists participated in this study. A total of 18 histological slides of biopsy, endoscopic submucosal dissection (ESD), and surgical resection of esophageal squamous lesions were diagnosed using a virtual slide system.

**Results:**

Most of noninvasive (intraepithelial) carcinomas diagnosed by Japanese pathologists were diagnosed as high- or low-grade dysplasia (intraepithelial neoplasia) or reactive atypia by the majority of Polish and German pathologists. Diagnoses of not only high-grade dysplasia but also low-grade dysplasia or reactive lesion by Western criteria were given for many biopsy specimens of cases in which the corresponding ESD or surgical specimens showed definite stromal invasion.

**Conclusion:**

There still exist differences in the histological diagnostic criteria for early stage esophageal carcinoma between Western and Japanese pathologists. The Japanese diagnostic criteria could improve agreement of diagnoses between biopsy and resected specimens of esophageal SCC. Moreover, diagnostic approaches using Western criteria may cause delay in the early diagnosis and treatment of esophageal SCC.

## Introduction

More than 10 years have passed, since the Vienna classification for gastrointestinal epithelial neoplasia was proposed in Gut [1]. It became clear during the consensus meeting at Vienna in Austria in 1998 that there have been considerable differences in the diagnostic histological criteria for early stage carcinoma between Western and Japanese pathologists. Namely, while stromal invasion is the most important diagnostic criterion of carcinoma for Western pathologists, the degree of nuclear and structural abnormality is more important for Japanese pathologists, regardless of the presence or absence of stromal invasion [1–5].

During the last decade, the differences in histological criteria have been debated and the need for the establishment of a unified approach to the practical diagnosis and treatment of gastrointestinal neoplasia has been highlighted [6–15].

Joint workshops consisting of Polish, Japanese, and some German pathologists were held in Poland in 2011 to clarify and review the present status of the histological criteria, i.e., Western and Japanese of early stage esophageal SCC, and also to clarify their significance and accuracy.

## Materials and methods

The histological slides of esophageal squamous cell lesions used were from the Japanese patients of Hitachi General Hospital in Hitachi City and of Shizuoka Prefectural Cancer Center in Shizuoka, Japan.

Esophageal squamous lesion materials consisted of 6 endoscopic biopsies, 4 ESD specimens, and 8 surgical specimens. Lesions of Barrett’s esophagus were not included in the present study.

The relationship between biopsies and ESD/surgically resected specimens in each case is shown in Table 1, but participating pathologists were blinded for the relationship before making diagnoses.

**Table 1:** Eight esophageal squamous epithelial lesions: relation between biopsies and ESD/surgical specimens

	Case	Site	End/macro^#^	Biopsy no.	ESD/surg^##^ (S) no.
Age/gender					
1	72/M	Mt	Reddish	Eso-B1	Eso-ESD2
2	65/M	Mt	0-IIc (+I)***	Eso-B2	Eso-ESD3
3	70/M	Mt	Flat, red	Eso-B3	Eso-S1&S2
4	59/M	ECJ	Irregular	Eso-B4	Eso-S3&S4
5	62/M	Mt	0-IIc***	Es0-B5	Eso-S5&S6
6	82/M	Mt	Flat	Eso-B6	Eso-ESD4
7	73/M	(−)	0-Is + IIb***	(−)	Eso-ESD1
8	66/M	Mt	0-IIa + IIc***	(−)	Eso-S7&S8

*Mt* middle thoracic esophagus, *ECJ* esophago-cardiac junction

*** Japanese macroscopic classification: 0-IIc: slightly depressed, I: Elevated, Is: Elevated, sessile, IIb: Flat, IIa: slightly elevated

^#^Endoscopic or macroscopic findings

^##^Endoscopic submucosal dissection/surgical specimen

Participating pathologists made their diagnoses of the presented cases by accessing the Virtual Slide System through the Internet. {Virtual Slides: NCC-CIR (National Cancer Center–Cancer Image Reference Database at the Homepage of the National Cancer Center, Tokyo, Japan) was used in 2011}.

The participating pathologists involved in diagnosis consisted of 20 Polish, 3 German, and 6 Japanese pathologists.

All the participating pathologists were equally provided with the same information of each specimen, such as location, color, and shape of the lesion, endoscopic pictures for biopsy specimens, and macroscopic pictures for ESD/surgical specimens.

Before making diagnoses, all the participants were requested to answer questionnaires concerning the diagnostic criteria that the participants used in daily diagnostic work, i.e., Western or Japanese criteria.

### Western histological criteria

The presence of stromal invasion of neoplastic cells is required for the diagnosis of esophageal SCC [4, 14, 16].

In this study, the following findings were regarded as stromal invasion in squamous cell lesions: (1) infiltration of neoplastic cells into the lamina propria mucosae or deeper layer; (2) lympho-vascular invasion; and (3) growth of neoplastic squamous cells in desmoplastic stroma.

### Japanese histological criteria

Degrees of cellular and structural abnormalities are more important for the diagnosis of esophageal SCC regardless of the presence of stromal invasion [1, 4, 11, 12, 17].

Features of cytological abnormality included: (a) variation in nuclear size and shape; (b) presence of markedly hyperchromatic, large nuclei; (c) irregularly clumped chromatin; (d) loss of nuclear (or cellular) polarity. Features of structural abnormality included: (e) irregular (disorganized) arrangement of atypical cells (loss of regular maturation toward the surface, and including front formation against normal cells). These features were regarded as histological criteria of intraepithelial SCC (cis).

In this study, noninvasive carcinoma is defined as intraepithelial SCC without apparent stromal invasion; cis.

Definite carcinoma is defined as SCC with apparent stromal invasion.

### Statistical analysis

All statistical tests were analyzed using the Chi-square test with Yates’ correction for the comparison of distribution of diagnoses.

A *p* value of less than 0.05 was considered to indicate a statistically significant difference. All statistical tests were completed using SAS version 9.4 (SAS Institute, Cary, NC, USA).

## Results

In answer to the questionnaires before the diagnosing the specimens, 18 (90%) of 20 Polish and all 3 German pathologists replied that their primary diagnostic criterion of esophageal SCC is the “presence of stromal invasion of neoplastic epithelium (or cells)”, while all the Japanese participants answered “nuclear atypia and/or architectural atypia regardless of stromal invasion”.

Concerning the operation of the virtual slide system, 7 (24%) out of 29 participants (including 3 German pathologists) complained of the difficulty in accessing the system as well as of slow reaction. The other participants mentioned no problems.

The results of the diagnoses made by participating pathologists are shown in Table 2.

**Table 2:** Distribution of diagnoses for (a) esophageal lesions’ total specimens and (b) esophageal lesions’ biopsy specimens

Diagnoses	Polish: 20 pathologists		German: 3 pathologists		Japanese: 6 pathologists	
	No. of cases	%	No. of cases	%	No. of cases	%
(a) Esophageal lesions total specimens						
1. Reactive/regenerative	17	4.7	5	9.8	0	0
2. Indefinite for neoplasia	7	1.9	1	2.0	0	0
3. Low-grade dysplasia	14	3.9	5	9.8	3	2.8
4. High-grade dysplasia	63	17.5	13	25.5	2	1.9
Subtotal (benign)	101	28.1	24	47.1	5	4.6
5. Susp. of carcinoma	10	2.8	2	3.9	2	1.9
6. Noninvasive carcinoma	67	18.6	6	11.8	37	34.3
7. Carcinoma in dysplasia	16	4.4	6	11.8	0	0
8. Definite carcinoma	93	25.8	11	21.6	51	47.2
9. Carcinoma (SM~)	73	20.3	2	3.9	13	12
Subtotal (malignant)	259	71.9	27	52.9	103	95.4
Total number (%)	360	100	51^a^	100	108	100

(b) Esophageal lesions biopsy specimens						
1. Reactive/regenerative	17	14.2	5	27.8	0	0
2. Indefinite for neoplasia	7	5.8	1	5.6	0	0
3. Low-grade dysplasia	13	10.8	4	22.2	3	8.3
4. High-grade dysplasia	26	21.7	5	27.8	2	5.6
Subtotal (benign)	63	52.5	15	83.3	5	13.9
5. Susp. of carcinoma	7	5.8	1	5.6	2	5.6
6. Noninvasive carcinoma	24	20	2	11.1	20	55.6
7. Carcinoma in dysplasia	7	5.8	0	0	0	0
8. Definite carcinoma	16	13.3	0	0	9	25
9. Carcinoma (SM~)	3	2.5	0	0	0	0
Subtotal (malignant)	57	47.5	3	16.7	31	86.1
Total number (%)	120	100	18	100	36	100

*Reactive/Regenerative* reactive lesion or regenerative lesion,* Carcinoma* (SM~) carcinoma with invasion to submucosal or deeper layer

^a^Someone did not make a diagnosis for three specimens

Among the total number of 18 histological slides, 71.9/52.9% were diagnosed as malignant lesions {including (5) suspicious of carcinoma, (6) noninvasive carcinoma ~ (8) definite carcinoma and (9) carcinoma (SM~) in Table 2a} by Polish/German pathologists as opposed to 95.4% by Japanese pathologists. Conversely, benign diagnoses by Polish/German pathologists were made in 28.1/47.1%, but only in 4.6% by Japanese (*p* < 0.001).

The diagnosis of high-grade dysplasia was remarkably higher by Polish/German pathologists than by Japanese, 17.5/25.5 vs 1.9% (*p* < 0.001 for Polish and German vs Japanese).

The discrepancy in frequency of diagnoses between Polish/German and Japanese pathologists is even more clearly seen in the diagnoses of biopsy specimens than in diagnoses for all specimens (Table 2). Namely, 47.5/16.7% of the biopsy specimens were diagnosed as malignant by Polish/German pathologists, compared with 86.1% by Japanese (*p* < 0.001). Conversely, benign diagnoses by Polish/German pathologists were made in 52.5/83.3%, but only in 13.9% by Japanese (*p* < 0.001). The difference of the frequency of malignant diagnoses between Polish pathologists and Japanese pathologists was 23.5% (95.4–71.9%) in case of the total specimen (Table 2) and 38.5% (86.1–47.5%) in case of biopsy specimens (Table 2) (23.5 < 38.5%). The same tendency was seen between German pathologists and Japanese pathologists (42.5 < 69.4%; 95.4–52.9, 86.1–16.7, Table 2)

As shown above, many biopsy specimens diagnosed as ‘noninvasive carcinoma’ by Japanese pathologists were diagnosed as high-grade dysplasia, low-grade dysplasia, indefinite for neoplasia, or reactive/regenerative lesions by Polish and German pathologists.

## Case presentation

Esophageal case 1 consisted of biopsy B1 and ESD2 specimens from the middle of the thoracic esophagus (Table 1) showing the representative distribution of diagnoses by both Western and Japanese histological criteria (Table 3).

**Table 3:**
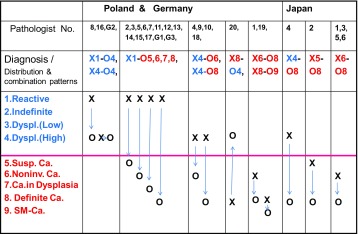
Esophagus Case 1: distribution and combination patterns of diagnoses: biopsy (B1: X )–ESD2 (O), Poland: 1–20, Germany: G1–3, Japan: J1–6

This table shows distribution and combination “patterns” of diagnoses

*Reactive/Regenerative* reactive lesion or regenerative lesion,* Carcinoma* (SM~) carcinoma with invasion to submucosal or deeper layer, *Dyspl. (Low)* low grade dysplasia, *Dyspl. (High)* high grade dysplasia, *Susp. Ca* suspicious of carcinoma, *Noninv. Ca* noninvasive carcinoma, *X-O* represents just “combinationpattern” of diagnoses between biopsy (X) and corresponding ESD (O) specimens, but not the “exact number” of the combination itself

**Fig. 1:**
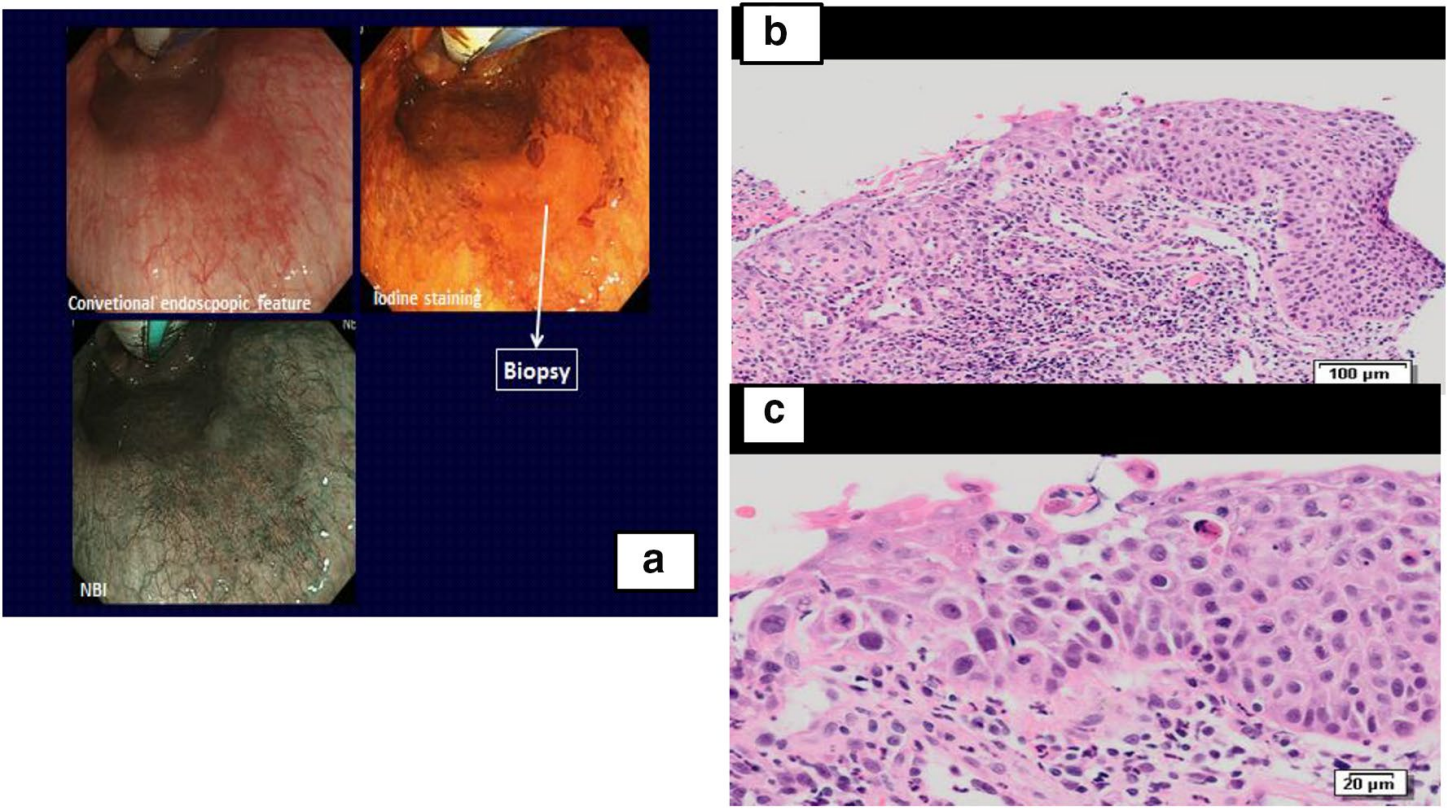
Case 1: Endoscopy and biopsy (B1). **a** *Left* Endoscopy: conventional endoscopy examination reveals *reddish* mucosa in the *middle* thoracic esophagus which was unstained by endoscopic iodine spray and *brownish* on a *narrow* band imaging (NBI) endoscopy. **b** *Right top* biopsy histology (B1): a bird’s eye view. Whole squamous epithelium including basal layer is replaced by high cellular atypical cells with hyperchromatic nuclei. **c** *Right bottom* higher power view of biopsy B1: polarity of the basal layer cells is lost. Irregular arrangement of nuclei and several large and hyperchromatic nuclei are seen

**Fig. 2:**
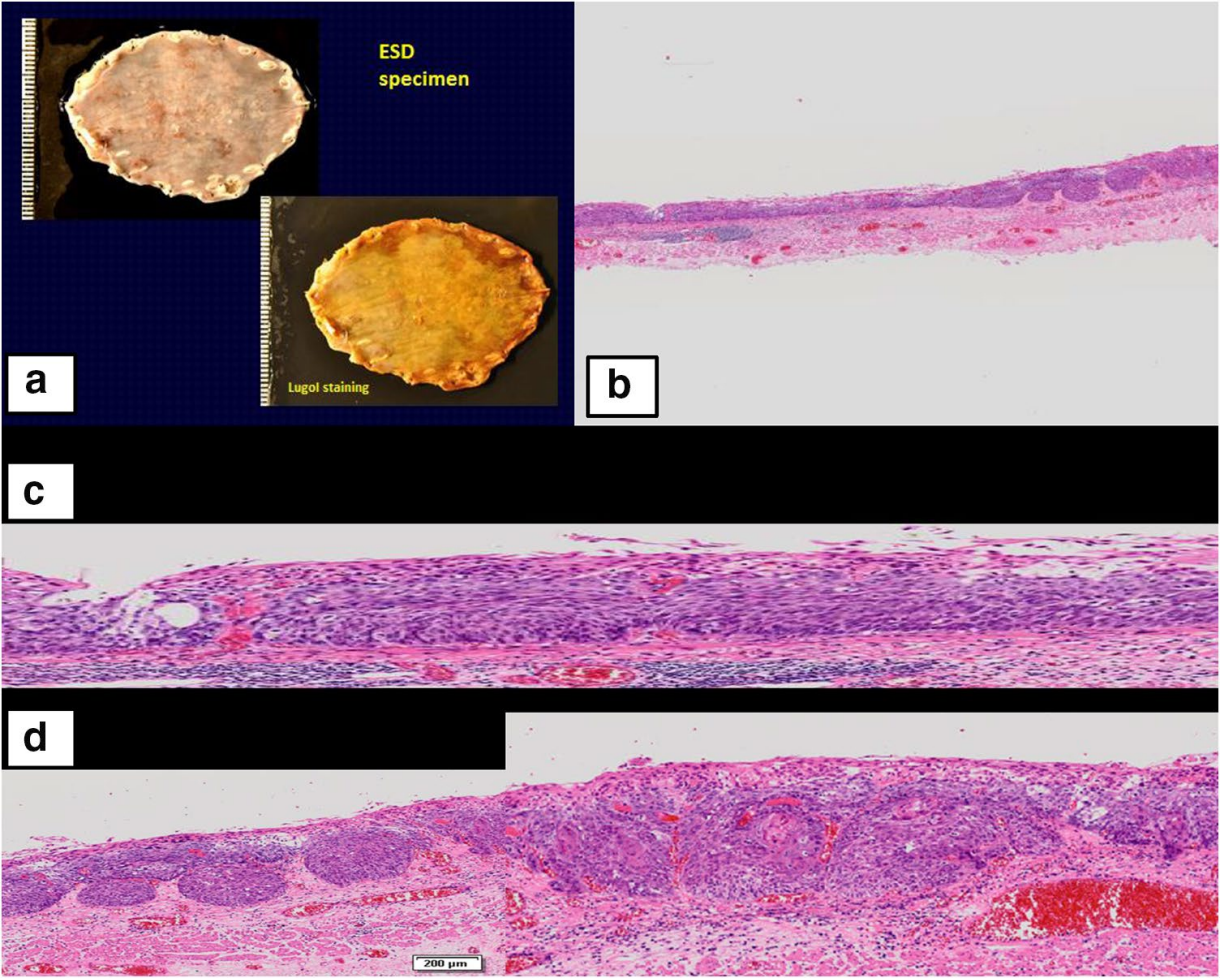
Case 1: ESO-ESD2 and its histology. **a** Gross findings of ESD specimen (ESO-ESD2): *Upper* a slightly irregular and granular mucosal pattern is seen in the formalin-fixed specimen. *Lower* a wide unstained area is detected in the iodine-stained specimen. **b** *Right top* a bird’s eye view of the section #6 of ESD2 specimen. **c** *Middle* a higher power view of the *left* side of the section #6 showing the extension of noninvasive carcinoma (cis; irregular arrangement of neoplastic squamous cells with loss of nuclear polarity). **d** *Bottom* a higher power view of the *right* side of the section #6 showing the invasion of squamous cell carcinoma to the lamina propria mucosae

Figure 1a–c shows the endoscopic findings and histology of the biopsy from case 1. In addition, Fig. 2a–d shows macroscopic findings of ESD2 and its histological findings.

Biopsy B1 was diagnosed as malignant by 5 of 6 Japanese pathologists, but only by 3 of 20 Polish pathologists. One Japanese and 5 Polish pathologists diagnosed the lesion as high-grade dysplasia, while the others diagnosed reactive atypia of squamous epithelium (Table 3).

All 6 Japanese, 17 Polish, and 2 German pathologists diagnosed the ESD 2 specimen histologically as definite carcinoma (with stromal invasion) or suspected carcinoma. A larger discrepancy between the diagnoses of B1 and ESD 2 was seen in the diagnoses by Polish and German pathologists than in the diagnoses by Japanese pathologists (Table 3) (*p* = 0.019).

However, 3 Polish pathologists used the term noninvasive carcinoma and/or definite carcinoma for B1, following the same histological criteria as the Japanese ones (Table 3).

Summarizing all the cases, there were 5 esophageal cases in which ESD or surgical specimens showed a definite stromal invasion {3 or more Japanese pathologists and 3 or more Polish (and/or German) pathologists gave diagnoses of definite carcinoma, or carcinoma with invasion to the submucosal layer, for the ESD or surgical specimens}.

Table 4 shows the distribution of biopsy diagnoses for the 5 esophageal invasive carcinomas.

**Table 4:** Distribution of biopsy diagnoses in cases of which ESD/surgical specimen showed definite stromal invasion—esophageal lesions

Case no.	Japanese: 6 pathologists. Distribution of biopsy diagnoses				Polish and German: 23 pathologists. Distribution of biopsy diagnoses				Total*
	Ca^a^ (Noninv^b^)	HD^c^	LD^d^	R/I^e^	Ca (Noninv)	HD	LD	R/I	
Case 1	5 (4)	1	0	0	3 (1)	5	0	15	23
Case 2	6 (3)	0	0	0	15 (14)	5	1	2	23
Case 3	6 (3)	0	0	0	13 (12)	8	0	2	23
Case 4	6 (5)	0	0	0	15 (11)	7	1	0	23
Case 5	6 (4)	0	0	0	13 (13)	5	2	2	22**
Total (%)	29 (29/30:97%)	1 (1/30:3%)			59 (59/114:52%)	30 (30/114:26%)	4 (4/114:4%)	21 (21/114:18%)	114**/114: (100%)

* Total number of Polish and German pathologists who participated in diagnosing

** There was one pathologist who did not make diagnosis for case 5

^a^Carcinoma

^b^Noninvasive carcinoma

^c^High-grade dysplasia

^d^Low-grade dysplasia

^e^Reactive lesion or indefinite for neoplasia

Japanese pathologists diagnosed the biopsies of 5 esophageal invasive carcinomas as carcinoma (including noninvasive carcinoma) in 97% (29/30), and high-grade dysplasia (HD) in 3% (1/30), while Polish and German pathologists diagnosed the biopsy specimens as carcinoma in 52%, HD in 26%, low-grade dysplasia (LD) in 4%, and reactive lesion/indefinite for neoplasia (R/I) in 18%. Polish and German pathologists diagnosed biopsy specimens of about a half (48%) of the esophageal invasive carcinoma as benign (Table 4). There are statistical differences for the diagnosis of HD or LD or R/I between Poland/Germany in 48% (55/114) and Japan in 3% (1/30) (*p* < 0.001), and also for the diagnosis of LD or R/I between Poland/Germany in 22% (25/114) and Japan in 0% (0/30) (*p* = 0.045).

In case of early stage carcinoma of the esophagus, greater diagnostic discrepancies between biopsy and resected specimens were seen when using Western histological criteria than when using Japanese criteria. Diagnoses of not only high-grade dysplasia but also low-grade dysplasia, reactive lesion, or indefinite for neoplasia by Western criteria were given to many biopsy specimens of cases in which the corresponding ESD or surgical specimens showed definite stromal invasion.

Concerning the procedures or treatments for those patients after the biopsies is diagnosed:

In Japan, most of patients with biopsy’s diagnosis of squamous carcinoma in situ (intraepithelial carcinoma according to Japanese criteria) undergo Endoscopic Submucosal Dissection (ESD) or Endoscopic Mucosal Resection (EMR) after endoscopic and radiological evaluation for cTNM of the disease.

Some cases may be treated by chemo-radiation therapy following patient’s selection.

In Poland, in case of high-grade dysplasia (according to western criteria), most of them are followed up with consecutive biopsies, and only cases with definite carcinoma (i.e., with stromal invasion) and/or cases for which obligatory two separate pathologists make a same diagnosis of high-grade dysplasia/neoplasia are treated by surgical operation (for example, partial esophagectomy). ESD or EMR is performed only for a limited number of patients in a few specialized hospitals.

In case of low-grade dysplasia, generally, they are all followed up with repeated biopsies (with an interval from 3 to 6 months).

In case of regenerative atypia, patients are treated against the lesion as benign, and thereafter, they have a biopsy with longer interval from 6 to 12 months. All patients with esophageal dysplasia have a radiological examination for cTNM (personal information by AN-G and EZ-N).

## Discussion

The distribution of the diagnoses for esophageal lesions (Table 2) showed that many lesions diagnosed as noninvasive carcinoma or definite carcinoma by Japanese pathologists were diagnosed as not only high-grade dysplasia but also low-grade dysplasia, or even as merely reactive lesions by Polish and German pathologists using Western histological criteria. The differences in diagnoses between Polish/German and Japanese pathologists were clearly caused by the basic differences in definitions as well as histological criteria of carcinoma primarily used by both groups. Namely, it basically depends on whether they accept the concept of noninvasive (intraepithelial) carcinoma or not.

Furthermore, many lesions diagnosed according to Western criteria as high-grade dysplasia, low-grade dysplasia, or reactive atypical lesions for biopsy showed stromal invasion in some areas of the corresponding ESD or surgical specimens (Tables 3, 4).

It is clear that a greater discrepancy in diagnoses between biopsy and ESD/or surgical specimens was seen in pathologists who followed Western criteria than in pathologists who followed Japanese criteria.

However, there were some Polish pathologists who accepted the concept of noninvasive carcinoma and used the term in the workshops, although many of them answered in the questionnaires that they used the Western histological criteria in their routine diagnostic work.

The reason for the greater discrepancy of diagnoses between biopsy and ESD/surgical specimens with Western criteria is that biopsy only samples the superficial (namely, epithelial) layer of the lesion; therefore, it often does not contain information from the deeper layer of the lesion, where stromal invasion as well as submucosal invasion may be present. Consequently, it is often impossible to establish a diagnosis of carcinoma for biopsy specimens when following Western criteria, whereas it is possible to make the diagnoses of carcinoma when Japanese criteria are followed, since they take into account nuclear or architectural abnormality or both, regardless of stromal or submucosal invasion of neoplastic cells. Several papers have been reported about the difference in histological criteria between Western and Japanese pathologists for gastrointestinal tumors [1–5]:

Schlemper and his associates reported the differences between Western and Japanese pathologists from 1997 to 2000 [1–4] and suggested that there was a greater discrepancy between the results of biopsy and ESD/surgical specimens when using Western criteria than when using Japanese criteria [2]. However, they mainly focused on proposing a new international consensus classification for gastrointestinal epithelial neoplasia (Vienna classification).

Stolte and his associates also reported significant discrepancies between biopsy-based and resected specimen-based diagnoses, which they ascribed to diagnostic inexperience on the part of Western pathologists [6, 10].

Diagnosis of intraepithelial neoplasia, squamous cell carcinoma in situ, and early invasive carcinoma are often difficult for Western pathologists because of mild architectural and cytological abnormalities. Squamous cell carcinoma in situ sometimes resembles low-grade intraepithelial neoplasia (/dysplasia), which was pointed out by Arai et al. in their introduction of Japanese viewpoint of histological diagnosis for early stage of esophageal carcinoma [18]. Actually, as shown by the results of the present study, many diagnoses of “low-grade dysplasia or reactive changes” according to Western criteria were given for the biopsies of esophageal neoplasias which showed stromal invasion in the lamina propria mucosae or to the submucosal layer in the corresponding ESD/surgical specimens (Tables 3, 4).

The authors also know that there are many Western pathologists who understand “High-Grade Intraepithelial Neoplasia (HGIN)”, or High-Grade Dysplasia (HGD) by Western criteria is almost equivalent to carcinoma in situ (or noninvasive carcinoma, or intraepithelial squamous carcinoma) by Japanese criteria, and that such Western pathologists have been increasing in number, since Vienna classification was proposed in 2,000.

There are somewhat differences between Poland and Japan in the processes which patients with high-grade dysplasia undergo as described in the result. In Poland, it may take a longer time to determine the final diagnosis of malignancy and the treatment for the patients is mainly surgical operation (e.g., partial esophagectomy) which is very invasive for a patient. Therefore, Polish pathologists and clinicians tend to wait until the biopsy shows invasive carcinoma. However, the difference between the two countries may be regarded as not so large in case of high-grade dysplasia, because the lesion is removed either by surgical or by endoscopic treatment.

However, in case of low-grade dysplasia, or reactive atypia by Western criteria, it may take a longer time in Poland than in Japan to confirm that the lesion is actually invasive carcinoma, and then, the risk of the lesion to develop up to advanced carcinoma becomes high. This is to be avoided.

The present study demonstrated that biopsy’s findings of high-grade dysplasia, low-grade dysplasia, and reactive atypia according to Western criteria already include the risk of the presence of invasive carcinoma in the same lesion. In other words, Japanese histological criteria of intraepithelial carcinoma/or CIS may be regarded as histological findings which act as a most dependable marker for the risk of accompanying invasive carcinoma.

To clarify the difference in diagnostic criteria for reactive/regenerative, indefinite for neoplasia, low (or high)-grade dysplasia, and intraepithelial carcinoma, between Western and Japanese pathologists, Table 5 is presented.

**Table 5:** Comparison of diagnoses and histological criteria of esophageal squamous intraepithelial lesions between Western and Japanese pathologists

Diagnoses	Western histological criteria^#^	Japanese histological criteria^##^
Reactive/regenerative	No or slight cellular atypia, Cellular maturation toward the surface preserved	The same as those of Western criteria However, if a, b, and/or c findings (below) are present, the lesion is diagnosed as intraepithelial carcinoma*
Indefinite for neoplasia	Between reactive and LGD	Between reactive and LGD
Low-grade Dysplasia (LGD)	Atypical (primitive/basaloid) cells in lower 1/2 of the epithelium Disorganization of epithelium and/or loss of cell polarity may be present	LGD: Atypical (primitive/basaloid) cells in lower 1/2 of the epithelium* a. Marked variation in nuclear size and shape ➡ Intraepithelial carcinoma b. Loss of cell polarity, marked disorganization➡ Intraepithelial carcinoma c. Markedly hyperchromatic and large nuclei ➡ Intraepithelial carcinoma
High-grade Dysplasia (HGD)	Atypical (primitive/basaloid) cells in more than lower 1/2 of the epithelium Greater crowding, Loss of cell polarity, Disorganization of epithelium	HGD: Atypical (primitive/basaloid) cells in more than lower 1/2 of the epithelium* a. Marked variation in nuclear size and shape ➡ Intraepithelial carcinoma b. Loss of cell polarity, marked disorganization ➡ Intraepithelial carcinoma c. Markedly hyperchromatic and large nuclei ➡ Intraepithelial carcinoma
Noninvasive carcinoma (CIS or intraepithelial carcinoma)	None available, in most text books However, in some textbooks**, these nomenclatures and histological criteria as the same as Japanese are accepted	Markedly disorganized arrangement of atypical cells with lack of surface maturation, and/or with any of a, b, and c: a. Marked variation in nuclear size and shape b. Loss of cell polarity c. Markedly hyperchromatic and large nuclei

* According to Japanese histological criteria (as common consensus), a, b, and c are key findings of intraepithelial carcinoma, even though the surrounding epithelium is similar to HGD, LGD, or reactive/regenerative lesions

** References: [19, 20]

^**#**^Summarized from [14, 16, 19]

^**##**^Personal information (with common consensus, MI)

In addition, three pictures as supplementary materials are added to show the key histological findings which strongly suggest the risk of accompanying invasive carcinoma.

Takubo et al. insisted that the term “carcinoma in situ” should be used instead of high-grade dysplasia/intraepithelial neoplasia to describe an intraepithelial neoplasm that is histologically and cytologically similar to the intraepithelial spreading component of an invasive carcinoma [11].

Shimizu et al. reported the clinical and pathologic features of esophageal early squamous cell carcinoma and pointed out the necessity of having a consensus meeting between Japanese and Western pathologists as well as endoscopists to reach a firm common ground for nomenclature [13]. These results and their suggestions support the results and the conclusion of our present study.

Following the present Western criteria may prevent opportunities for early diagnosis and treatment of an enormous number of esophageal cancer patients in the future.

This is an urgent problem that should not be difficult to solve.
